# PAPP-A as a Potential Target in Thyroid Eye Disease

**DOI:** 10.1210/clinem/dgae339

**Published:** 2024-05-16

**Authors:** Cheryl A Conover, Laurie K Bale, Marius N Stan

**Affiliations:** Division of Endocrinology, Mayo Clinic, Rochester, MN 55905, USA; Division of Endocrinology, Mayo Clinic, Rochester, MN 55905, USA; Division of Endocrinology, Mayo Clinic, Rochester, MN 55905, USA

**Keywords:** thyroid eye disease, PAPP-A, insulin-like growth factor, proinflammatory cytokines, fibrocytes

## Abstract

**Context:**

Proptosis in thyroid eye disease (TED) can result in facial disfigurement and visual dysfunction. Treatment with insulin-like growth factor I receptor (IGF-IR) inhibitors has been shown to be effective in reducing proptosis but with side effects.

**Objective:**

To test the hypothesis that inhibition of IGF-IR indirectly and more selectively with PAPP-A inhibitors attenuates IGF-IR signaling in TED. Informed consent was obtained from patients with TED undergoing surgery, and retro-orbital tissue was collected for fibroblast isolation and culture. Operations were performed in Mayo Clinic operating suites. Cell culture was performed in a sterile tissue culture facility. Retro-orbital tissue was collected from 19 patients with TED.

**Methods:**

Treatment of TED fibroblasts with proinflammatory cytokines. Flow separation of CD34^−^ and CD34^+^ orbital fibroblasts, the latter representing infiltrating fibrocytes into the orbit in TED. PAPP-A expression and proteolytic activity, IGF-I stimulation of phosphatidylinositol 3 kinase/Akt pathway, and inhibition by immuno-neutralizing antibodies against PAPP-A, CD34^+^ status, and associated PAPP-A and IGF-IR expression were measured.

**Results:**

Proinflammatory cytokines markedly increased PAPP-A expression in TED fibroblasts. IGF-IR expression was not affected by cytokine treatment. Inhibition of PAPP-A's proteolytic activity suppressed IGF-IR activation in orbital fibroblasts from patients with TED. TED fibroblasts that were CD34^+^ represented ∼80% of the cells in culture and accounted for ∼70% of PAPP-A and IGF-IR–expressing cells.

**Conclusion:**

These results support a role for PAPP-A in TED pathogenesis and indicate the potential for novel therapeutic targeting of the IGF axis.

Thyroid eye disease (TED), also known as Graves ophthalmopathy or thyroid-associated orbitopathy, is the most serious extrathyroidal complication that develops in 25% to 50% of patients with autoimmune Graves hyperthyroidism (1-5). It is characterized by immune cell infiltration and orbital inflammation, expansion of orbital adipose tissue, enlargement of extraocular muscle, and accumulation of extremely hydrophilic glycosaminoglycans. The increased orbital tissue volume within the unyielding bony orbit results in the clinical hallmark of TED (ie, proptosis—the forward displacement of the eyeball). This can result in facial disfigurement and reduced quality of life with deleterious consequences for eye function. A better understanding of the pathological mechanisms underlying TED may identify novel targets to inhibit the development and progression of TED before any clinical manifestation.

Loss of immune tolerance to the thyroid-stimulating hormone receptor (TSHR) and generation of activating antibodies against the protein are central to the thyroidal pathology of Graves disease. The presence of TSHR on orbital fibroblasts from patients with TED implicates it in extrathyroidal pathology as well. Moreover, pivotal studies revealed a physical and functional interaction between the TSHR and insulin-like growth factor-I receptor (IGF-IR) that is essential in promoting TED pathogenesis (6-8).

##  

### Insulin-Like Growth Factor Receptor

IGF-IR mediates the effects of IGF ligands on cell proliferation, differentiation, and survival. IGF-IR was found to be overexpressed in orbital tissue from patients with TED (9, 10). Indeed, IGF-IR inhibitors attenuated signaling from either TSHR or IGF-IR in cultured TED fibroblasts (6, 7, 11-13). These findings led to the use of inhibitory monoclonal antibodies against IGF-IR as a therapeutic option for TED (14, 15). Teprotumumab, which binds to and blocks IGF-IR signaling, was recently FDA-approved for treatment of TED (7, 16, 17). Clinical trials in patients with active disease showed benefit of this inhibitory monoclonal antibody on the reduction of proptosis. Recent trials also suggested efficacy in patients with inactive, stable TED (18). However, adverse events have been documented with teprotumumab likely due to the ubiquitous nature of IGF-IR and its interactions with insulin signaling (19). A better understanding of IGF signaling and its regulation in orbital tissue is needed to fully understand the mechanism and avoid/reduce off-target consequences. One approach would be to target modifiers of IGF-IR signaling, instead of the IGF-IR per se. We propose PAPP-A as such a target.

### PAPP-A

PAPP-A is a novel zinc metalloprotease that can increase pericellular IGF bioavailability through specific cleavage of inhibitory IGF binding proteins, in particular IGFBP-4 (20, 21). PAPP-A is a secreted protein that tethers to the surface of cells through heparan sulfate–like proteoglycan moieties in an autocrine/paracrine fashion. IGF bound to IGFBP-4 is unable to activate receptors. However, upon cleavage of IGFBP-4 by PAPP-A, IGF is liberated from the complex in the pericellular environment and IGF-IR signaling is initiated. PAPP-A–induced enhancement of local IGF-I action through proteolysis of IGFBP-4 has been demonstrated in several in vitro systems and appears to serve a similar function in vivo (22). Conversely, inhibition of PAPP-A expression or its proteolytic activity represents an innovative approach to decrease IGF availability with resultant attenuation of IGF-IR signaling with minimal side effects. Studies in PAPP-A–deficient mice and use of specific pharmacological inhibitors of PAPP-A–mediated IGFBP-4 proteolysis in vivo underscore the health benefit and safety profile of PAPP-A inhibition in various disorders (21, 23-28).

The most potent stimulators of PAPP-A expression in many cell types are the proinflammatory cytokines tumor necrosis factor (TNF)-α and interleukin (IL)-1β, which are elevated in orbital tissue of patients with TED (9, 29, 30). Of note, TED fibroblasts may have an exaggerated response to various stimuli, including proinflammatory cytokines, and inflammatory signaling potentiates TED fibroblast proliferation and differentiation into adipocytes (1, 15, 29).

### Orbital Fibroblasts

Excessive activity of orbital fibroblasts is at the core of TED (10). Orbital fibroblasts comprise a heterogenous population of cells possessing divergent phenotypes and differentiation potential (31). In addition, circulating CD34^+^ fibrocytes/precursor cells coming from the bone marrow infiltrate the orbit in TED (32). There is increased generation of fibrocytes in TED (33). These cells transition to CD34^+^ orbital fibroblasts and occupy the orbit that otherwise contains CD34^−^ fibroblasts. CD34^+^ fibroblasts express high levels of IGF-IR (33). They are not generally found in healthy orbital tissue or in dermal fibroblast cultures, implicating enhanced IGF-IR signaling in CD34^+^ orbital fibroblasts in the pathology of TED.

IGF-I's proproliferative and proadipogenic activity in orbital fibroblasts from patients with TED could be a major driver of pathogenesis (34). We propose that inhibition of IGF-IR indirectly and more selectively with PAPP-A inhibitory antibodies will be effective and with fewer side effects (35, 36). To generate preclinical support for this hypothesis we used primary cultured cells and showed that (1) PAPP-A is expressed in orbital fibroblasts from patients with TED, (2) proinflammatory cytokines stimulate PAPP-A expression, (3) inhibition of PAPP-A's proteolytic activity reduces IGF-I signaling in orbital fibroblasts from patients with TED, and (4) CD34^+^ orbital fibroblasts represent 80% of cells in culture from patients with TED, and approximately 70% of these cells were accountable for PAPP-A and IGF-IR expression. These results support a role for PAPP-A in TED pathogenesis and suggest novel therapeutic targeting of the IGF axis.

## Materials and Methods

### Materials

Cell culture reagents were purchased from Gibco (Medium 199, fetal bovine serum [FBS], penicillin/streptomycin and L. glutamine). Gentamicin was purchased from Sigma and all flasks and plates were purchased from Falcon. IL-1β, TNF-α, IL-6, soluble IL-6 receptor, IGF-I, and IGFBP-4 were from R&D systems.

### Patient Samples

Table 1 presents the sex, age, Clinical Activity Score (CAS—measure of TED inflammatory state), proptosis indication, thyroid-stimulating hormone receptor antibodies (TRAbs), thyroid-stimulating immunoglobulins (TSIs), smoking status, and duration of disease of 19 deidentified patients with TED from which we isolated fibroblasts from retro-orbital fat during operations from 2018 to 2021. Informed consent was obtained as approved by the Institutional Review Board of the Mayo Clinic. On the day of surgery, orbital tissue was removed and placed in sterile transport medium for pick-up from the operating room. Samples were processed immediately under aseptic conditions for cell culture in Media 199 containing 20% fetal bovine serum, glutamine, and antibiotics, as described previously (37, 38). At 90% confluency, aliquots of cells were frozen at −80 °C.

**Table 1.** Patients with TED

Patient no.	Sex	Age at surgery	CAS preop	Indication for orbital decompression	Smoking status	TRAb preop	TSI preop	Excess proptosis (mm)*^a^*	TED duration to surgery (months)
TED-001	m	64	6	Proptosis, symptomatic	0	na	3.6	3	1
TED-002	f	62	3	Proptosis	0	na	6.6	7	16
TED-003	m	59	1	Proptosis	0	2.1	4.4	4	12
TED-004	f	62	0	Proptosis, symptomatic	0	1.4	na	5	96
TED-005	m	77	1	Proptosis	0	0.5	na	8	120
TED-006	f	63	2	Proptosis	0	3.5	na	4	13
TED-007	m	73	4	DON	0	4.1	na	0	6
TED-008	f	57	4	Globe subluxation	0	na	na	5	60
TED-009	m	66	3	DON	0	14	7.1	5	15
TED-010	m	60	0	Proptosis	0	38	4.6	14	84
TED-011	m	47	1	DON	2	2.1	3.2	8	20
TED-012	m	46	3	Proptosis	0	1.4	5.3	6	42
TED-013	f	37	0	Proptosis, symptomatic	2	1.2	4.9	5	144
TED-014	f	67	1	Proptosis, symptomatic	0	4.9	na	6	242
TED-015	m	66	4	DON L + bilat proptosis	0	21	2.1	9	13
TED-016	m	55	1	Proptosis, symptomatic	0	4.3	4.1	6	16
TED-017	f	32	0	Proptosis, symptomatic	0	na	4.9	2	17
TED-018	f	42	0	Proptosis, symptomatic, corneal exposure	0	0.5	na	5	84
TED-019	f	56	4	DON	0	7.2	4.2	8	9

Informed consent was given by these patients to obtain retro-orbital fat from decompression surgery.

Smoking status—0 = never smoked or quit >1 year ago; 1 = quit within the last year; 2 = active smoker.

Abbreviations: CAS, Clinical Activity Score—7-point scale, score ≥ 3 indicates active disease; DON, dysthyroid optic neuropathy; na, not available; TED, thyroid eye disease; TRAb, thyroid-stimulating hormone receptor antibody; TSI, thyroid-stimulating immunoglobulin.

^*a*^Maximum excess value entered if both orbits were decompressed.

### Cell Culture

Orbital adipose tissue samples were sterilely minced with surgical scissors, digested in collagenase, filtered, washed, and plated, as described previously (34, 39). Orbital fibroblasts were cultured in Media 199 containing 10% FBS. For experiments, cells were used between passage 3 and 7. At ∼90% confluency, fibroblasts were washed and changed to Media 199 containing 0.1% FBS without and with experimental additions. At 24 hours, cells were harvested for PAPP-A and IGF-IR mRNA expression. At 72 hours, conditioned medium (CM) was collected for determination of PAPP-A protein and IGFBP-4 proteolysis. Results from the 72-hour CM were normalized to culture cell number. Cells were cultured in batches of 4 TED samples (male, female; low CAS, high CAS). A total of 12 different fibroblast cultures from patients with TED were studied in 3 separate sets as follows: TED-007, -010, -018, -019; TED-001, -004, -008, -011; TED-005, -006, -015, -017.

### RNA Isolation and Reverse Transcriptase-Quantitative Polymerase Chain Reaction

Total RNA was extracted as previously described (39, 40). Briefly, cells were treated with Trizol (Ambion Life Technologies, Carlsbad, CA) and further processed as per manufacturer's instruction. RNA (1 μg) was reversed transcribed with the SuperScript III Frist-Strand Synthesis System (Life Technologies) and evaluated by quantitative real-time polymerase chain reaction (PCR) using the CFX Connect Real-Time System with iTAQ Universal SYBR Green Supermix (Bio-Rad, Hercules, CA). Amplification plots were analyzed using CFX Maestro Software version 4.1 (Bio-Rad). Amplified PCR products were purified through QIAquick Gel Extraction Kit (Qiagen Hilden, Germany), quantified and serial diluted from 10^8^ to 10^3^ molecules. Relative quantification and fold changes were based on the standard curve for each gene. Primers hPAPP-A: forward: TGACCGTGCGTGACATCCC; reverse: GCAAAAGGCTCGGTTGTTGAT. Primers hIGF-IR: forward: CCCAAATTATGTGTTTCCGAAAT; reverse: CCAGGTTATGATGATGCGATTCT.

### PAPP-A Enzyme-Linked Immunosorbent Assay

PAPP-A levels in the CM were measured using an ultrasensitive human PAPP-A enzyme-linked immunosorbent assay kit (RRID:AB_2783656) from Ansh Labs (Webster, TX).

### IGFBP-4 Protease Assay

Recombinant IGFBP-4 (10 ng) was added to cell-free CM samples which were then incubated without or with addition of IGF-II (50 ng) for 24 hours at 37 °C, as previously described (20, 39). To confirm specificity, precomplexed IGFBP-4/IGF-II was added to CM that had been preincubated for 1 hour at 37 °C with a specific neutralizing monoclonal antibody generated against a unique exosite in PAPP-A (mAb-PA 1/41) (RRID:AB_2861192) that is required for IGF-dependent IGFBP-4 proteolysis (35, 36). This was then incubated for 24 hours at 37 °C. IGFBP-4 proteolysis was assessed by Western blotting using a TAG antibody generously provided by Professor Claus Oxvig (Aarhus University, Denmark) and horseradish peroxidase–conjugated secondary antibody (RRID:AB_2337942) followed by electrochemiluminescense. Blots were visualized on a Li-Cor Odyssey imager.

### Flow Cytometry

TED orbital fibroblasts (∼5 × 10^6^ cells) were stained with CD34/PE/581 (BioLegend #343505) (RRID:AB_1731937) for 30 minutes at 4 °C. Washed cells were sterile-sorted using an Aria 4 Laser in the Microscopy and Cell Analyses Core at Mayo Clinic Rochester. Human dermal fibroblasts were used as a negative control.

### IGF-IR Signaling

TED orbital fibroblasts were grown to confluency in Media 199/10% FBS. Cells were washed and medium changed to 0.1% FBS with 10 nM TNF-α for 24 hour to induce PAPP-A expression. Without changing medium, 10^−7^ M mAb-PA1/41 was added to the medium 30 minutes prior to the addition of precomplexed 10 nM IGF-I/20 nM IGFBP-4. Cells were harvested after 15 minutes and lysed in 150 μL of RIPA buffer (0.15 M NaCl, 0.5% NP-40, 0.1% SDS, 50 mM Tris pH 7.6) with phosphate inhibitors (2 nM sodium orthovanadate, 10 mM sodium pyrophosphate, 40 mM β-glycerol phosphate, 10 mM sodium fluoride, protease tablet) and sonicated for 5 seconds. Under reducing conditions, 32 μL of sample was separated by 7.5% Mini-PROTEAN TGX gel (BioRad Laboratories) and transferred to polyvinyl difluoride. Filters were blocked with 5% nonfat milk in Tris-buffered saline with 0.1% Tween 20 (TBS-T), and then incubated overnight at 4 °C with primary antibody (phosphorylated Akt [S473] [RRID:AB_329825], 1:1000 and total Akt [RRID AB_329827], 1:1000 [both from Cell Signaling Technology, Inc. Danvers, MA]). Membranes were then washed 3 times for 5 minutes in TBS-T and incubated with secondary antibody with a streptavidin–horseradish peroxidase conjugate (RRID:AB_2337942) and chemiluminescent substrate (Pierce, Rockford, IL) for detection.

### Statistical Analysis

Analysis of variance with post hoc Dunnett's was used when comparing multiple treatments to a single control (no treatment) or Tukey's for multiple comparisons. Western blottings for proteolytic assays are quantitative. Significance was set at *P* < .05. Data are expressed as mean ± SEM of triplicates from independent experiments, each involving cells from a different donor.

## Results

### Thyroid Eye Disease

The patients with TED who served for collection of the samples were all euthyroid at the time of the orbital decompression. Most had hyperthyroidism secondary to Graves disease (n = 18), but 1 patient had hypothyroidism related to Hashimoto thyroiditis. All had positive TSH receptor antibodies (either TRAb and/or TSI). There were 2 (10.5%) active smokers and 17 (89.5%) who quit more than a year previously or never smoked. The reason for orbital decompression was proptosis reduction for exposure pathology in patients with inactive disease (n = 10, 52.6%), proptosis reduction with inflammation unresponsive to medical therapy (n = 3, 15.8%), compressive optic neuropathy (n = 5, 26.3%), and globe subluxation (n = 1, 5.3%). The median excess proptosis predecompression value was 5 mm (range 0-14 mm above average for gender and ethnic group). In the subgroup of tissue samples that were cultured, there were 5 patients with inactive TED while the others had CAS ≥ 3.

### PAPP-A Expression in Orbital Fibroblasts From TED Patients: Regulation by Cytokines

**Figure 1.**
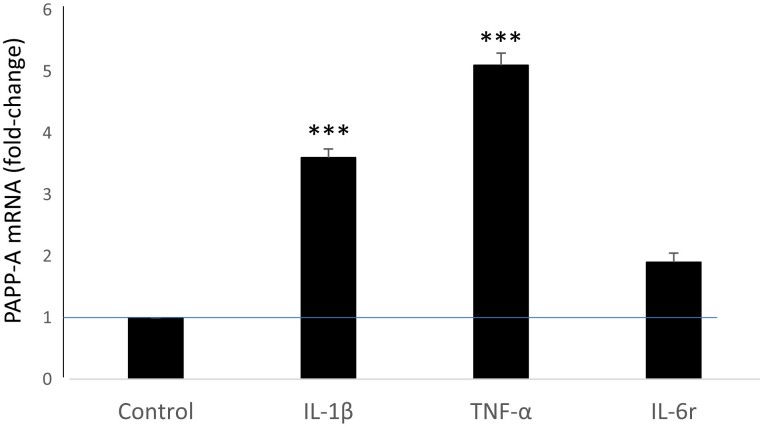
PAPP-A mRNA expression in orbital fibroblasts treated with the indicated pro-inflammatory cytokines for 24 hours. Results (fold change from untreated control) are mean ± SEM of 12 TED cultures. ***P < .0001.

**Figure 2.**
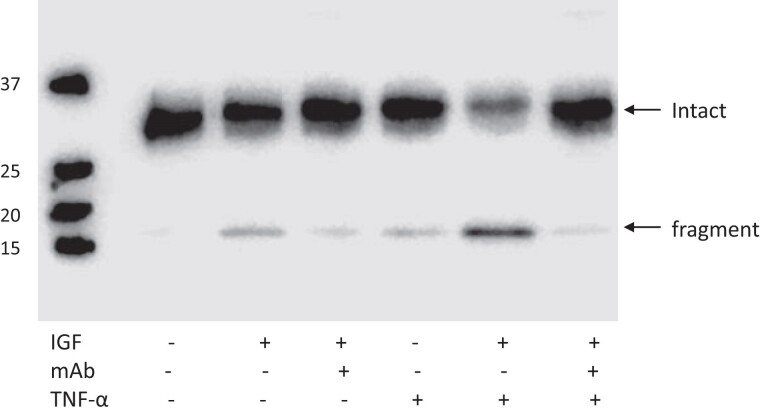
PAPP-A–mediated IGFBP-4 proteolysis. Representative image of cell-free IGF-dependent IGFBP-4 proteolysis in 72-hour conditioned medium from TED fibroblasts without and with the indicated additions. All lanes had IGFBP-4 added. Molecular weight markers are indicated on the left. Arrows indicate intact and fragmented IGFBP-4.

Primary orbital fibroblasts from patients with TED were treated with IL-1β, TNF-α, and IL-6 with its soluble receptor (IL-6r), and had RNA isolated after 24 hours for RT-qPCR (Fig. 1). PAPP-A expression was increased ∼4-fold over control (no treatment) with IL-1β and TNF-α (*P* < .0001). IL-6r treatment of TED fibroblasts had little effect on PAPP-A expression levels. There were no differences in response of TED fibroblasts obtained from male and female subjects, and the extent of expression was not related to the patient's CAS. There was no effect of cytokine stimulation on IGF-IR expression (fold-change: 1.0 ± 0.05, 1.0 ± 0.05, 1.5 ± 0.44 for IL-1β, TNF-α, IL-6r, respectively). Secreted PAPP-A protein levels in 72-hour CM paralleled mRNA expression, namely 4 ± 0.4-fold with IL-1β treatment and 3 ± 0.3-fold with TNF-α treatment (*P* < .0001). IL-6r had no effect. PAPP-A secreted by TED fibroblasts (n = 4) was active as an IGF-dependent protease (7 ± 2.3-fold, IGF-II vs control) with near complete inhibition by mAb-PA1/41. TNF-α treatment alone increased proteolysis 5 ± 1.0-fold, which was further increased by IGF-II another 6 ± 0.7-fold. A representative protease assay is shown in Fig. 2. Thus, TED fibroblasts in culture express active PAPP-A that is markedly increased with proinflammatory cytokine treatment.

In subsets of CD34^−^ and CD34^+^ fibroblasts isolated from the parental culture, CD34^+^ represented 82 ± 3%; (mean ± SEM, n = 12) of fibroblasts from TED cultures. 68 ± 3% of PAPP-A expression and 68 ± 6% of IGF-IR expression by TED fibroblasts were associated with CD34^+^ cells.

### Effect of PAPP-A Inhibition on IGF-IR Activation

**Figure 3.**
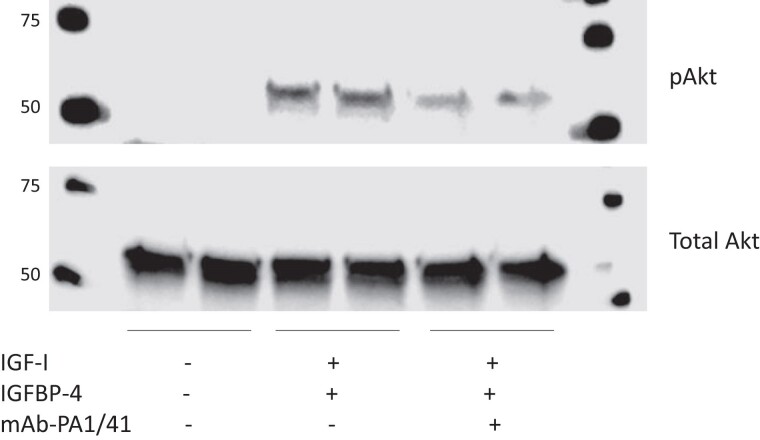
Representative Western blot for phosphorylated Akt (pAkt) and total Akt without and with the indicated additions. Molecular weight markers are indicated on the left.

IGF-I stimulation of the PI3 K/Akt signaling pathway mediates cell proliferation, differentiation, and survival (41). TNF-α-induced PAPP-A expression in TED fibroblasts enhanced IGF-I/IGFBP-4 stimulated IGF-IR activation of the PI3 K/Akt pathway, as evidenced by increased phosphorylated pAKT (Fig. 3). There was no effect on total Akt. Importantly, inhibitory mAb-PA1/41 reduced IGF-I's effects on receptor activation by 53 ± 4%, n = 5 (*P* < .0001).

## Discussion

In this study we used primary cultures of retro-orbital fibroblasts to determine regulation of PAPP-A expression and its effects on IGF-IR signaling in TED. The key findings were that (1) fibroblasts from patients with TED express and secrete proteolytically-active PAPP-A; (2) proinflammatory cytokines, IL-1β and TNF-α, markedly increased PAPP-A mRNA and protein expression in fibroblasts from patients with TED; IGF-IR mRNA expression was not changed with any treatments; (3) inhibition of PAPP-A's proteolytic activity suppressed IGF-IR activation in orbital fibroblasts from patients with TED; and (4) CD34^+^ orbital fibroblasts represented ∼80% of cells in TED and comprised ∼70% of the cells expressing PAPP-A and IGF-IR. These data support our hypotheses that PAPP-A plays a significant role in promoting TED and that targeted inhibition of PAPP-A could be a therapeutic option or adjuvant.

All the samples were collected from euthyroid individuals and smoke exposure was minimal, thus eliminating these known risk factors for TED deterioration from impacting the ongoing TED pathophysiology at the time of samples collection.

TNF-α and IL-1β were potent stimulators of PAPP-A mRNA and protein expression in TED fibroblasts. There were no significant differences in induction of PAPP-A expression with sex or CAS status. Three patients were operated on due to active disease, and 6 patients were operated on due to sight-threatening disease, also consistent with active and progressive disease. The other 10 patients were operated on during the inactive and stable phase of the disease. By design, we used maximal concentrations of cytokines in these cell culture models. Proinflammatory cytokines in TED are more highly expressed in active vs stable disease (32), and, therefore, patients with active TED may show higher levels of PAPP-A in orbital tissue in vivo. IGF-IR expression was not affected by cytokine treatment, indicating that PAPP-A was a major driver of IGF-IR activation in TED. PAPP-A expressed by fibroblasts from patients with TED was proteolytically active against IGF-bound IGFBP-4. Thus, in combination with it being accessible at the cell surface, PAPP-A is an attractive target for immuno-neutralizing antibodies against PAPP-A–mediated IGFBP-4 proteolysis (35, 36). The specificity of this approach comes from the fact that the orbital inflammatory milieu is the one driving the expression of PAPP-A and thus its blockade would have little effect on other organs (8). IL-6, which is a prominent cytokine in TED, did not affect PAPP-A expression. The reasons for this are unclear but may have to do with different regulatory pathways.

CD34^+^ fibroblasts derived from circulating fibrocytes/precursor cells have been reported to promote TED pathology (32, 33). Indeed, we found that the vast majority of fibroblasts from TED were CD34^+^. This cell type appeared to account for ∼70% of PAPP-A and IGF-IR expression. There is considerable fibroblast heterogeneity in orbital tissues (42), and it is likely that other cells besides CD34^+^ contributed to PAPP-A expression. However, the expression of PAPP-A and IGF-IR in CD34^+^ fibroblasts could explain, at least in part, the specificity for enhanced IGF-IR activity in TED.

IGF-I stimulates proliferation and adipogenesis pathways in TED fibroblasts (10). Here we show that TNF-α-induced PAPP-A enhances IGF-I–stimulated PI3 K/Akt, a downstream signaling pathway of IGF-IR in proliferation, adipogenesis, and cell survival (41). Moreover, this phosphorylation was significantly inhibited by mAb-PA1/41 indicating that PAPP-A is an important regulator of IGF-I signaling.

These results provide rationale for developing PAPP-A as therapeutic target in TED (43). We have shown effectiveness of inhibitory mAb-PA1/41 in vitro and in vivo (25, 27, 35, 39, 44). There are major advantages to targeting PAPP-A proteolytic activity: (1) it is an enzyme of unique amino acid sequence lending itself to specificity; (2) it is secreted and associated with the extracellular surface of cells and therefore drug-able; (3) its expression is both condition and cell specific and therefore selective; (4) its inhibition would result in moderate restraint of IGF signaling; and (5) side effects are expected to be minimal, since mice with PAPP-A gene deletion show healthy longevity with no secondary endocrine or metabolic dysfunction (21, 23-26). Additionally, combination therapy with IGF-IR antibodies, FcRn inhibitory monoclonal antibodies, or with other drugs under development using different pathways would allow for lower doses of both agents with a likely improved side-effect profile and possible synergistic efficiency.

There are limitations to this study. First, the sample numbers are small. Nevertheless, the responses of fibroblasts from patients with TED were consistent and marked. Second, there are the inherent limitations of cell culture, which cannot recreate the interactive processes that occur in vivo. Nevertheless, the results support a role for PAPP-A in TED. Also, we have not directly addressed effects of PAPP-A/IGF in TED fibroblasts on regulation of hydrophilic glycosaminoglycan production in this study. However, there have been several studies reporting that IGF-I regulates hyaluronan synthesis in TED fibroblasts (14, 45) but not in fibroblasts from control subjects (46). These effects of IGF-I on proliferation, adipogenesis, cell survival, and hyaluronan production, enhanced by PAPP-A, would be expected to contribute considerably to increased orbital volume in TED.

**Figure 4.**
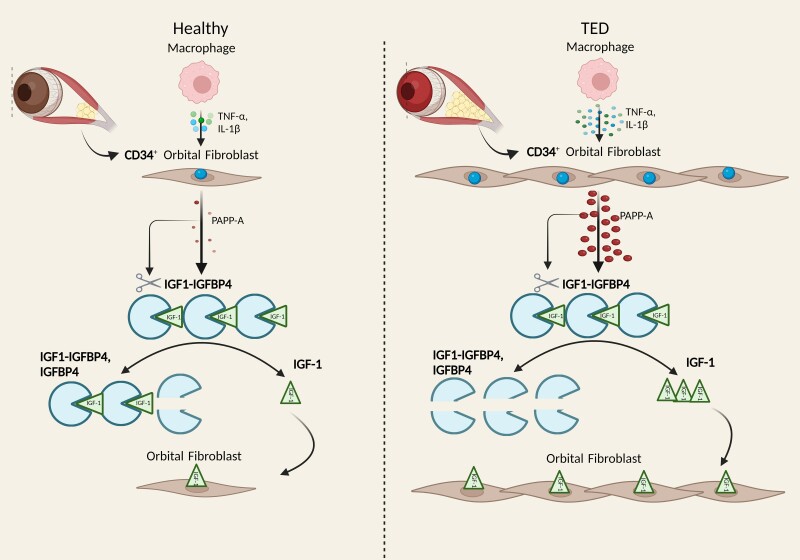
Graphic representation of the role of PAPP-A in TED.

In conclusion, these data indicate that proinflammatory cytokines in the orbital tissue of TED stimulates CD34^+^ fibroblasts to express and secrete proteolytically active PAPP-A that enhances local IGF-IR activity. This activity could be inhibited by a specific monoclonal antibody that inhibits PAPP-A–mediated IGFBP-4 proteolysis (Fig. 4). Thus, these results support a role for PAPP-A in TED pathogenesis and indicate the potential for novel therapeutic targeting of the IGF axis.

## Data Availability

Data supporting the results in this paper will be peer-reviewed. No large datasets were generated during the study that would require archiving in a repository.

## Abbreviations

CAS: Clinical Activity Score
CM: conditioned medium
FBS: fetal bovine serum
IGF-IR: insulin-like growth factor I receptor
IL: interleukin
PCR: polymerase chain reaction
TED: thyroid eye disease
TNF: tumor necrosis factor
TRAb: thyroid-stimulating hormone receptor antibody
TSHR: thyroid-stimulating hormone receptor
TSI: thyroid-stimulating immunoglobulin
